# Atmospheric methane removal: a research agenda

**DOI:** 10.1098/rsta.2020.0454

**Published:** 2021-11-15

**Authors:** Robert B. Jackson, Sam Abernethy, Josep G. Canadell, Matteo Cargnello, Steven J. Davis, Sarah Féron, Sabine Fuss, Alexander J. Heyer, Chaopeng Hong, Chris D. Jones, H. Damon Matthews, Fiona M. O'Connor, Maxwell Pisciotta, Hannah M. Rhoda, Renaud de Richter, Edward I. Solomon, Jennifer L. Wilcox, Kirsten Zickfeld

**Affiliations:** ^1^ Department of Earth System Science, Stanford University, Stanford, CA 94305-2210, USA; ^2^ Woods Institute for the Environment, and Precourt Institute for Energy, Stanford University, Stanford, CA 94305-2210, USA; ^3^ Department of Applied Physics, Stanford University, Stanford, CA, USA; ^4^ Department of Chemical Engineering and SUNCAT Center for Interface Science and Catalysis, Stanford University, Stanford, CA, USA; ^5^ Department of Chemistry, Stanford University, Stanford, CA, USA; ^6^ SLAC National Accelerator Laboratory, Stanford University, Stanford, CA, USA; ^7^ Global Carbon Project, CSIRO Oceans and Atmosphere, Canberra, Australian Capital Territory 2601, Australia; ^8^ Department of Earth System Science, University of California at Irvine, Irvine, CA 92697, USA; ^9^ Mercator Research Institute on Global Commons and Climate Change, Berlin, Germany; ^10^ Geographisches Institut, Humboldt Universität zu, Berlin, Germany; ^11^ Met Office Hadley Centre, FitzRoy Road, Exeter EX1 3PB, UK; ^12^ Department of Geography Planning and Environment, Concordia University, Montreal, Quebec, Canada; ^13^ Chemical and Biomolecular Engineering Department, University of Pennsylvania, Pennsylvania, PA, USA; ^14^ Ecole Nationale Supérieure de Chimie de Montpellier, Montpellier, Languedoc-Roussillon FR, USA; ^15^ Department of Geography, Simon Fraser University, Burnaby, British Columbia, Canada V5A 1S6

**Keywords:** methane oxidation, negative emissions, Methane Removal Model Intercomparison Project, iron salt aerosols, solar photocatalysts, zeolites

## Abstract

Atmospheric methane removal (e.g. *in situ* methane oxidation to carbon dioxide) may be needed to offset continued methane release and limit the global warming contribution of this potent greenhouse gas. Because mitigating most anthropogenic emissions of methane is uncertain this century, and sudden methane releases from the Arctic or elsewhere cannot be excluded, technologies for methane removal or oxidation may be required. Carbon dioxide removal has an increasingly well-established research agenda and technological foundation. No similar framework exists for methane removal. We believe that a research agenda for negative methane emissions—‘removal' or atmospheric methane oxidation—is needed. We outline some considerations for such an agenda here, including a proposed Methane Removal Model Intercomparison Project (MR-MIP).

This article is part of a discussion meeting issue 'Rising methane: is warming feeding warming? (part 1)'.

## 1. Introduction

The concentration (i.e. mole fraction) of methane (CH_4_) in the atmosphere continues to rise. The 14.7 ppb average global increase observed in 2020 was the largest of the past four decades [1]. Since 1750, its relative concentration has increased twice as fast as that of carbon dioxide (CO_2_) and is now more than 2.5 times pre-industrial levels [1]. Methane is the second most important anthropogenic greenhouse gas after CO_2_; the radiative forcings attributable to its direct (0.64 W m^−2^) and direct-plus-indirect effects (0.97 W m^−2^) are 38% and 58%, respectively, of the 1.68 W m^−2^ for CO_2_ [2].

**Figure 1.**
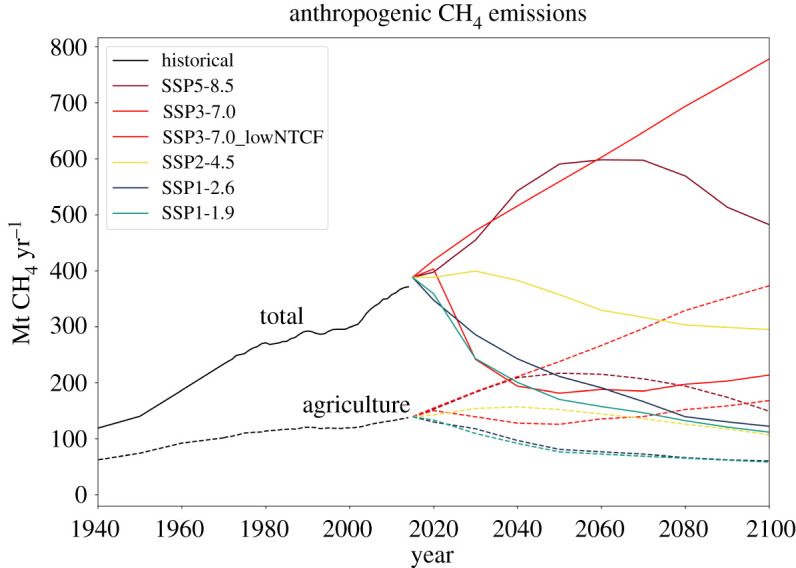
Global anthropogenic CH_4_ emissions (Mt CH_4_ yr^−1^) for the recent past and up to 2100 following the SSP emissions scenarios. Black lines show historical estimates from Hoesly *et al*. [3]; coloured lines show future projected emissions under the SSP marker scenarios [4]. Solid lines denote anthropogenic total emissions, whereas dashed lines show emissions from agriculture alone. Data available from https://tntcat.iiasa.ac.at/SspDb (accessed 11 May 2021). (Online version in colour.)

Global methane emissions approached a record 600 Tg CH_4_ yr^−1^ in 2017 (figure 1, precise estimates and uncertainties shown in table 1), with anthropogenic sources contributing 61% of the total (approx. 365 Tg CH_4_ yr^−1^; [5–7]). The global total for 2017 was 50 Tg CH_4_ yr^−1^ more than the average for the period 2000–2006, primarily because anthropogenic emissions were 13% higher. Agriculture-related sources in 2017 contributed approximately two-thirds of global anthropogenic methane emissions (227 Tg CH_4_ yr^−1^) and fossil fuels contributed the other third (108 Tg CH_4_ yr^−1^), with a smaller contribution from biomass burning (28 Tg CH_4_ yr^−1^) [5].

**Table 1.** Global methane emissions in 2017. Values are given in Tg CH_4_ yr^−1^ with minimum and maximum estimates in brackets (from data in [5,7]).

total	anthropogenic	agriculture	fossil fuels	biomass burning
596 (572–614)	364 (340–381)	227 (205–246)	108 (91–121)	28 (25–32)

As the dominant global source of anthropogenic methane, agricultural emissions are attributable primarily to cattle, sheep, and other ruminants, rice farming, and managing manures and waste. Methane emissions from agriculture continue to rise (figure 1), driven by global increases in total and *per capita* meat consumption as global population and wealth grow [8]. A number of technological and behavioural changes can, and likely will, reduce methane emissions substantially [9,10].

Reaching zero methane emissions in global food production appears particularly unlikely this century (figure 1). Dietary supplements such as essential oils and red algae can reduce methane emissions from individual cattle and sheep, but they sometimes do so at the expense of feed digestion and fermentation efficiency [11]. For rice farming, a meta-analysis of 52 studies found that non-continuous flooding reduced methane emissions by 53% on average compared to continuously flooded paddies; however, nitrous oxide (N_2_O) emissions increased by 105% and yield decreased by 4% [12]. Agricultural activities also dominate anthropogenic emissions of N_2_O [13] along with those of CH_4_ [14].

Methane emissions associated with the extraction, distribution, and use of fossil fuels grew by one-sixth from the early 2000s to 2017 [5,7]. New satellite, drone and other image-based approaches are helping to find and reduce fossil-fuel-related emissions (e.g. [15]). Although perhaps not as intractable as eliminating agricultural emissions, eliminating all fugitive and other emissions associated with energy extraction and use also seems difficult, unless fossil fuel consumption were to end entirely [16].

Along with difficulties in reaching zero methane emissions from agriculture and fossil fuel use, Earth-system feedbacks could rapidly increase methane emissions from natural systems [17,18]. Potential methane release from permafrost systems in the East Siberian Arctic Shelf (ESAS) is one possibility. This concern arises from suggestions that rapid methane release 55 million years ago at the boundary of the Paleocene and Eocene epochs triggered temperature increases of 5–8°C globally [18]. A recent study in the nearshore environment of the ESAS showed that ice-bonded permafrost had retreated 14 cm yr^−1^ over the past three decades [19]. Such subsea permafrost degradation or loss of coastal methane clathrates could lead to bursts of methane reaching the atmosphere, depending on water depth and other factors. Although recent shipborne and latitudinal analyses of methane emissions do not suggest that increased emissions from Arctic systems have begun [5,7,20], a future scenario of accelerated methane release is possible [21,22].

Atmospheric methane removal may be needed to offset continued methane release and limit the global warming contribution of this potent greenhouse gas. Eliminating most anthropogenic methane emissions is unlikely this century, and sudden methane release from the Arctic or elsewhere cannot be excluded, so technologies for negative emissions of methane may be needed. Carbon dioxide removal (CDR) has a well-established research agenda, technological foundation and comparative modelling framework [23–28]. No such framework exists for methane removal. We outline considerations for such an agenda here. We start by presenting the technological considerations for methane removal: energy requirements (§2a), specific proposed technologies (§2b), and air processing and scaling requirements (§2c). We then outline the climate and air quality impacts and feedbacks of methane removal (§3a) and argue for the creation of a Methane Removal Model Intercomparison Project (§3b), a multi-model framework that would better quantify the expected impacts of methane removal. In §4, we discuss some broader implications of methane removal.

## 2. Technological aspects of methane removal

### (a) Energy requirements of methane removal

We first compare and contrast aspects of CH_4_ and CO_2_ removal. In contrast to CO_2_, CH_4_ can be oxidized catalytically, without the need for capture, in a thermodynamically favourable reaction: CH_4_ + 2O_2_ → CO_2_ + 2H_2_O (ΔH_r_ = −803 kJ mol^−1^), although such a reaction is difficult at typical conditions of atmospheric temperature and pressure [29]. Because of methane's potency as a greenhouse gas (34 times higher Global Warming Potential (GWP) than CO_2_ on a century timescale and 86 times higher on a 20-year timescale, [30]), considerably less methane removal is needed to realize the same climate impact. In fact, methane concentrations could in principle be restored to preindustrial levels (approx. 750 ppb) by removing approximately 3.2 of the 5.3 Gt CH_4_ currently found in the atmosphere [31], though methane could only be maintained at preindustrial levels by continuous removal that at least balanced anthropogenic methane emissions, currently 0.36 Gt CH_4_ yr^−1^ (table 1). This amount is orders of magnitude lower than annual anthropogenic emissions of carbon dioxide, which are currently approximately 40 Gt CO_2_ [32]. Some disadvantages of removing CH_4_ compared with CO_2_ are its relative scarcity in the atmosphere (200 times less abundant) and its lack of a quadrupole moment or weak acidity that, in the case of CO_2_, can be exploited for concentration and capture.

This relative scarcity of methane in the atmosphere leads to a higher minimum energy requirement for methane removal compared to CO_2_. The goal in a system meant to separate methane from air is to isolate dilute methane from the ambient air and separate it into a higher purity stream that can later be oxidized (forming CO_2_ and H_2_O) or used. We consider the absolute minimum thermodynamic work to separate species that must be provided to a system, given that it is reversible, isothermal and isobaric. This value depends on the inlet purity, outlet purity and per cent capture of the system, resulting in a logarithmic rather than linear relationship with inlet concentration. The empirical formula for this is given by Minimum Thermodynamic Work of Separation [33,34]

2.1 $$\begin{array}{rl} W_{\text{min}} & =RT(n_{B,s}\ln(y_{B,s})+n_{B-s}\ln(y_{B-s}))+RT(n_{c,s}\ln(y_{C,s})+n_{C-s}\ln(n_{C-s}))\\ & \;-RT(n_{A,s}\ln(y_{A,s})+n_{A-s}\ln(y_{A-s})), \end{array}$$

where *R* is the ideal gas constant, *T* is the temperature in *K*, $n_{i,s}$ is the molar flow rate of a specific species in a stream, *i*, which can be either A, B or C, representing the inlet, species-rich and exhaust streams, respectively, $n_{i-s}$ is the molar flow rate of the stream not containing the specific species, $y_{i,s}$ is the mole fraction of the specific species in a stream and $y_{i-s}$ is the mole fraction of the stream not containing the specific species.

In the case of capturing CO_2_ from the atmosphere at an average concentration of 410 ppm with a capture fraction of 70% and an outlet purity of 97% CO_2_, the minimum work is approximately 20.2 kJ mol^−1^ CO_2_ [34]. The energy requirement decreases as the concentration of CO_2_ increases. For example, the CO_2_ concentration of a natural gas exhaust stream is often 3–5% (30 000–50 000 ppm). Given the same capture fraction and outlet purity, the minimum work of CO_2_ capture would be 8–9 kJ mol^−1^ CO_2_ [34].

Here, we estimate the minimum work of separation for methane in a generic process. The inlet concentration of CH_4_ is assumed to be the average atmospheric concentration, approximately 1.88 ppm [1], approximately 200 times more dilute than atmospheric CO_2_. The increased dilution results in a minimum work of separation of nearly 33.5 kJ mol^−1^ CH_4_, at the same capture fraction of 70% and outlet purity of 97% CH_4_ that we used for CO_2_. This minimum work of separation is 60% higher for methane than for CO_2_ in the atmosphere, meaning that the minimum energy per mole removed for a methane removal system is 60% higher than for a CO_2_ removal system. However, because of the higher radiative forcing of methane, removing one mole from the atmosphere has a greater short-term climate impact than removing one mole of CO_2_.

**Figure 2.**
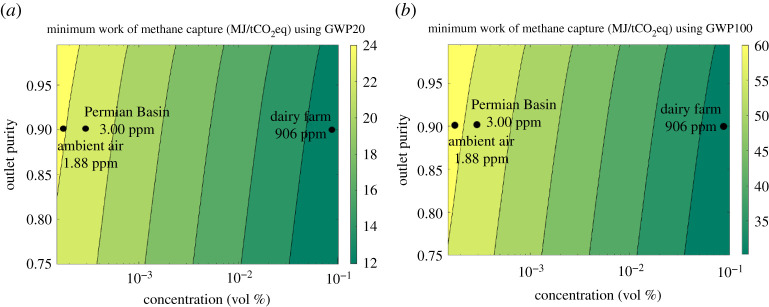
Minimum work of methane capture, holding capture fraction constant at 70% for different locations, the ambient air, the Permian Basin (as an example of a slightly higher 3 ppm CH_4_ case), and a dairy farm, corresponding to various concentrations, 1.88 ppm, 3.00 ppm and 906 ppm, respectively, normalized to MJ/tCO_2_eq using (*a*) GWP20 value of 86 and (*b*) GWP100 value of 34. (Online version in colour.)

To illustrate the higher radiative forcing associated with methane, the minimum work is normalized to a measure of MJ per ton of CO_2_ equivalent (MJ/tCO_2_eq) using a global warming potential over a 20-year time horizon (GWP20) of 86 and over a 100-year time horizon (GWP100) of 34. With the same capture fraction of 70% and outlet purity of 97%, the minimum work of methane capture is 24 MJ/tCO_2_eq and 62 MJ/tCO_2_eq using GWP20 and GWP100, respectively. For CO_2_, the minimum work of capture is 459 MJ/tCO_2_, meaning that the minimum work of capture for the same radiative forcing impact is 7 times lower using methane's GWP20 and 19 times lower using GWP100. Figure 2 shows how the minimum work of atmospheric methane capture varies with concentration and outlet purity, while holding capture fraction constant at 70%.

As the concentration of methane increases, the minimum work of separation decreases. This fact emphasizes that addressing higher concentration sources first is desirable, just as it is for CO_2_ removal, and that as more CH_4_ is captured directly from ambient air, further capture will require more work per unit CH_4_ removed as the background concentration drops. However, this may be remedied by strategically centring efforts of methane capture near regions where the methane concentration is consistently higher than in ambient air, including those associated with oil or natural gas extraction, abandoned coal mines, landfills and agriculture [35]. Technically, such efforts would be better described as methane mitigation because they target oxidizing elevated methane concentrations from known sources rather than methane at average concentrations in the bulk atmosphere. A dairy farm, where methane concentrations can be as high as 1000 times the average atmospheric concentration [36], is representative of a sector where methane emissions may be relatively difficult to eliminate, and methane oxidation could be strategic to employ. Avoiding methane emissions through local mitigation first—wherever possible—will almost always be less expensive and energy-intensive than methane removal from the bulk atmosphere.

### (b) Methane removal technologies

Atmospheric CO_2_ removal has a long history of research and a broad range of approaches studied [25,26]. Biological approaches for CO_2_ removal include reforestation/afforestation, soil carbon sequestration, biomass energy with carbon capture and storage and ocean iron fertilization; chemical or physical approaches include direct air capture of CO_2_ from ambient air, enhanced mineral weathering and enhanced ocean alkalinity (e.g. [27,37]). Some degree and type of CDR is included in all scenarios that keep average global surface warming below 1.5°C and most that keep it below 2°C [38], with many analyses examining the feasibility of removing approximately 10 Gt CO_2_ yr^−1^ [39–41], roughly one quarter of current total anthropogenic emissions [32].

In contrast to a well-established research community around negative emissions of CO_2_, the possibility of negative emissions for methane has been explored only relatively recently [31,42,43]. Soil-based agricultural approaches have a longer history of study (e.g. [44,45]), although often as mitigation from known sources rather than removal of methane from bulk air.

Here, we describe broad classes of technologies for methane removal, including photocatalysts, metal catalysts associated with zeolites and porous polymer networks, biological methane removal, including industrial approaches and approaches for managing soils in agricultural or other ecosystems, and iron-salt aerosol formation (table 2). For each of these technologies, research is needed on its cost, technological efficiency, scaling and energy requirements, social barriers to deployment, co-benefits and potential negative by-products. Research is also needed broadly on methane sorption to concentrate methane from low-concentration background air; having better sorbents would make methane removal technologies more efficient generally.

**Table 2.** Summary table of some methods for extracting methane from the atmosphere.

method	class	medium	air flow	sample references
photocatalysts	catalytic	substrate in air	active or passive	[46] Nature
zeolites or PPNs	metal catalysts	substrate in air	active or passive	[31] Nature Sustain.
iron-salt aerosols	physical	air	passive	[47] ESD
biotrickling filters	biological	substrate in air	active or passive	[48] Ecol. Eng.
soil amendments	biological	soil	passive	[44] Nutrient Cycling in Agroecosys.

Photocatalysts have the ability to oxidize methane and other hydrocarbons through heterogeneous (multi-phase) catalysis. One such catalyst is solid titanium dioxide (TiO_2_), a pigment used in paints and sunscreens. TiO_2_ is photocatalytically active when exposed to ultraviolet (UV) radiation [49] and can catalyse the same reaction that occurs when a flare burns methane:

2.2 $$\text{C}{\text{H}}_{4}+2{\text{O}}_{2}\to 2{\text{H}}_{2}\text{O}+\text{C}{\text{O}}_{2}.$$

In general, the reaction products desorb or release after their formation [46,50], eliminating the need for concentration and capture that occurs with geological carbon sequestration, but allowing molecules of the less potent greenhouse gas CO_2_ to enter the atmosphere [43]. Photocatalysts such as TiO_2_ can be applied in thin films to maximize surface contact with air. Silver-decorated zinc oxide (Ag-ZnO) nanocatalysts are another class of methane photo-oxidants; Chen *et al*. [46] coated ZnO semiconductors with Ag and documented a quantum yield of 8% at wavelengths less than 400  nm and greater than 0.1% at wavelengths of approximately 470  nm achieved for methane oxidation on the Ag-ZnO nanostructures.

**Figure 3.**
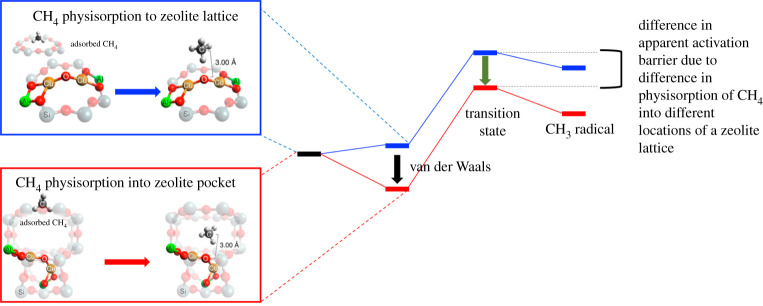
Comparison of two reaction coordinates for cleaving the strong H-C bond of CH_4_ (the first step in methane oxidation). The top (blue) reaction coordinate is for an H-atom abstraction of CH_4_ reacting with a Cu^II^-O-Cu^II^ active site that is exposed on the zeolite lattice. The bottom (red) reaction coordinate is for the same reaction as the top but with the Cu^II^-O-Cu^II^ active site located in a small pocket of a zeolite lattice. The strong physisorption of methane by the zeolite pocket (shown in the bottom box) lowers the apparent activation barrier for the reaction (top right (green) arrow shows the decrease in the transition state energy in red relative to blue reaction). (Online version in colour.)

Cu- and Fe-zeolites and porous polymer networks (PPNs) are two families of methane-oxidizing catalysts already of interest for converting methane to methanol (CH_3_OH), a partial oxidation product compared with fully oxidized CO_2_. Methanol has a much shorter lifetime than methane (weeks instead of years). Aluminosilicate zeolites have been well studied for the adsorption of CO_2_ from the atmosphere. Methane can also be concentrated from the atmosphere, but its interaction with the zeolite is weaker, on the order of approximately 5 kcal mol^−1^, as it relies only on van der Waals interactions with the oxygen atoms in the walls of the zeolite lattice; by contrast, CO_2_'s heat of adsorption can be as high as 14 kcal mol^−1^ given interactions with the CO_2_ quadrupole [51]. Scientists have screened more than 87 000 zeolite structures as potential methane sorbents [52]. After methane molecules are weakly bound by oxygen groups on the zeolite, Cu, Fe or other metal ions embedded on the lattice can oxidize the sorbed methane and release it as CO_2_ [31]. For specific zeolite topologies, the physisorption interaction with a constricted pocket in the lattice can contribute to lowering the apparent activation barrier in the catalysis of methane oxidation (figure 3) [53]. Relatively low-temperature methane oxidation for producing methanol has already been documented in zeolites such as Cu-ZSM-5 and Fe-ZSM-5, with Fe zeolites able to oxidize methane at room temperature [52,54]. Metal or other catalysts can also be deposited on, or embedded in, PPNs [55]. PPNs can be synthesized so that functional groups on the polymer backbone interact with methane in the micropores of the network. Higher temperatures and pressures lead to greater conversion efficiencies.

Microbes provide the second-largest natural sink for atmospheric methane of approximately 40 Tg methane annually [5]. At least two microbial groups, anaerobic archaea and aerobic methanotrophic bacteria, possess enzymes that oxidize methane: methane monooxygenase and methyl coenzyme M reductase [56]. These metalloenzymes also use Cu, Fe, or other metal sites for catalysis and have interesting parallels with the metallozeolites [57]. Efforts are underway to embed enzymes within three-dimensionally printed polymers that demonstrated promise in maintaining their stability [58]. Consortia of microbes have also been shown to couple the direct, anaerobic oxidation of methane to denitrification of nitrate, raising the potential of multi-gas mitigation [59]. Biologically mediated methane-to-methanol conversions are of particular interest in wastewater treatment systems where methanol is used to enhance denitrification rates [60].

Enhanced microbial oxidation of methane in agricultural and other soils or in artificial substrates (e.g. biotrickling filters) is a microbially based approach for methane mitigation or atmospheric removal (e.g. [44,45]). Han *et al*. [61] showed that amendments of biochar derived from rice straw reduced methane emissions from paddy soils by 40% in microcosm experiments, a case of methane mitigation from a known source (i.e. with elevated methane concentrations in air). The decrease was attributable to both decreased activity of methanogens and increased methane oxidation activity of methanotrophs. Sulfate additions have also been shown to reduce methane emissions from rice paddies [62]. Miller *et al*. [63] demonstrated that iron and humic acid amendments significantly suppressed *in situ* net methane fluxes by 26% in Arctic Alaska peatland soils, likely by enhancing alternative electron acceptor availability. This example is more analogous to methane removal from the bulk air because it was not associated with a known methane source.

Biotrickling filters have also been examined for methane removal from the atmosphere or methane mitigation from point sources such as landfills. Yoon *et al*. [64] modelled the feasibility of a biotrickling filtration system using methane-consuming bacteria to oxidize atmospheric methane. Their model indicated that atmospheric methane removal would be ineffective because the methane concentration is too low to enable cell survival. However, if concentrations were increased to 500–6000 ppmv CH_4_, similar to concentrations found near some landfills and concentrated animal feeding operations, 5 to 40 tons of methane could be mitigated per biofilter per year, depending on parameter assumptions in the model. Biocovers and biofilters containing methanotrophs are already used for methane mitigation from smaller or older landfills [48,65]. There is no *a priori* reason why biotrickling filters couldn't in principle be optimized for atmospheric removal more broadly.

Finally, researchers have proposed iron-salt aerosols (ISA) as a methane removal method [47,66]. ISA approaches attempt to convert methane into CO_2_ in the lower troposphere by enhancing natural sinks of the hydroxyl radical °OH (responsible for 90% of the natural methane sink) and the chlorine atom Cl (3–4% of the natural methane sink) [47]. This method mimics natural reactions associated with mineral dust particles in the atmosphere. Mineral dust contains iron, a micronutrient that can enhance ocean primary productivity and withdraw atmospheric CO_2_ [67,68]. Iron catalyses both °OH generation, through Fenton and photo-Fenton reactions in clouds and rain droplets [69,70], and Cl generation in sea salt aerosols [71,72]. The proposed method would enhance methane removal by releasing iron salt aerosols in the lower troposphere [47,66], increasing the Cl sink four-to-six-fold during the day and continuing to enhance the °OH sink at night. Cl atoms react with methane 16 times faster than °OH atoms do, and the iron(III)/iron(II) present in catalytic amounts could increase densities of Cl atoms provided by abundant sea-salt. ISAs have been invoked to explain why, just before the ice ages, the concentrations of both CO_2_ and methane decreased simultaneously; ice cores extracted from both Arctic and Antarctic poles show that there was 4–7 times more mineral dust during the glacial periods compared to the warmer interglacials [73].

### (c) Air processing and scaling requirements

Large volumes of air must be processed to realize Tg-scale conversion of methane to CO_2_. Air handling could pass through an initial step of adsorption, concentrating methane before contact with catalysts, radicals, or microbes (see above). Alternatively, active or passive systems could be used to remove methane directly at atmospheric concentrations. Electric fans powered by renewable fuels could be used to move the air in active systems [31,74], but research is needed to optimize rates of methane conversion against pressure drop through the system (e.g. [6,7,75]). By contrast, natural winds and air currents can provide air movement for passive removal systems; wind-based passive systems have been proposed for capturing CO_2_ based on anion-exchange and absorbent resins [76].

At larger scales, the generation of artificial wind for electricity generation has been proposed using solar updraft chimneys (SUC). These possibilities range from small ventilation systems for houses or buildings, inspired by Trombe walls [77], to full-scale 200–400 MW power plants generating carbon-free renewable energy [78]. A SUC uses a solar collector to warm air by the greenhouse effect; the hotter-than-ambient air then rises and a chimney enhances its speed by stack effect, turning turbines to generate electricity [78]. SUCs coupled with photocatalysts (activated by sunlight at ambient temperature) have been proposed as a removal method for methane [43], N_2_O [79] and halocarbons [80]. Photocatalysts coating the solar collector do not induce a pressure drop or energy loss, in contrast to active systems for CO_2_ capture or methane removal that may induce a pressure drop of approximately 100 Pa and a reduction of 20–25% of electricity output [81].

Regardless of the technology deployed, the volumes of air needed to be processed are substantial. At 15°C and 1013.25 hPa (abs), the density of air is 1.225 kg m^−3^. With a methane concentration of 1.88 ppm and a hypothetical conversion efficiency of 50%, the mass of air required to remove 1 Tg methane would be 2 × 10^6^ Tg, or 0.04% of the mass of the Earth's atmosphere. Such volumes are comparable in magnitude to those for direct air capture of CO_2_, where the scale of removal is Pg rather than Tg because the mass fraction of CO_2_ is 600 times greater. Studies are needed to evaluate aspects of scaling for all negative-emission technologies.

## 3. Impacts of methane removal

### (a) Climate impacts and feedbacks

We do not yet know whether or which methane removal technologies will prove commercially feasible at scale; further research and development are required for all of the examples we presented. We also argue that a better understanding of the climate and air quality benefits of methane removal is needed to enable a more complete cost-benefit analysis of the potential for methane removal. The potential atmospheric and Earth-system consequences of large-scale methane removal have complex feedbacks that need better quantification.

Methane removal clearly lowers atmospheric concentrations of methane, but the broader climate and air quality impacts of this removal will depend on methane's lifetime and how it is affected by changes in climate, methane concentration (e.g. the ‘methane-OH feedback factor'; [82]), and concentrations of other ozone precursors, among other factors. The latter two feedbacks are large, opposite in sign, and uncertain in pathways of rising methane and other ozone precursors [83], and will be important to quantify in scenarios of methane removal. The relationship between methane and tropospheric ozone is also uncertain and model-dependent (e.g. [84–86]) and requires close study to determine potential benefits of methane removal to surface air quality. Other Earth system feedbacks include the role of climate in accelerating the methane cycle [87] and direct effects of CO_2_ on methane emissions from wetlands as biosphere productivity is enhanced (e.g. [88]).

Modelling of methane interactions with climate, carbon cycle and air quality has to date drawn on simplified models, such as stand-alone land-surface schemes (e.g. [89]) or reduced-complexity models [90,91]. These results have shed light on important aspects of the system, including quantifying uncertainties and feedbacks [92,93] and the influence of methane feedbacks on climate targets [94]. Now that Earth System Models are emerging that combine fully coupled General Circulation climate models with interactive representations of land and ocean biogeochemistry, atmospheric chemistry and aerosols [85,87], coordinating the use of such models is important to address new questions such as the role of methane removal.

For scale, the current best estimates of the expected relationship between methane and temperature include: a 40% reduction in methane emissions by 2050 is predicted to cause a temperature reduction of 0.3°C [95], whereas a 2% annual reduction in methane concentration is predicted to reduce temperature by 0.5°C by 2100 [96]. More recently, Allen *et al*. [97] found that reductions in methane concentration can lead to a climate benefit even with reductions in aerosols resulting from strong air quality abatement measures. However, these studies were for methane mitigation rather than removal. The first study of removal, made possible with a methane emissions-driven model, found slightly larger temperature effects: a 40% reduction in methane emissions by 2050, for example, caused a temperature reduction of 0.4°C [85]. For carbon dioxide, positive emissions lead to a temperature response of slightly different magnitude than for the same quantity of negative emissions [98]; whether the same holds true for methane is unclear and will require emissions-driven modelling to address.

Climate and Earth-system impacts of methane removal extend beyond temperature reductions, including potential improvements in air quality through reduced human-induced changes in surface ozone concentrations (e.g. [84,85,97,99]). Surface ozone concentrations are directly linked to hundreds of thousands of premature deaths annually [100], and previous studies of the impact of methane reduction on surface ozone estimated that the marginal cost-effectiveness of each avoided premature mortality is approximately $US 400 000 [101]. Reduced ozone levels also increase net primary productivity of vegetation and crop yields [102].

Atmospheric chemistry modelling and experimentation are also needed to explore the potential consequences of unintended parallel reactions. Examples include the partial oxidation of methane to carbon monoxide (CO), methanol (CH_3_OH), or, for iron-salt aerosols, chloromethane (CH_3_Cl) instead of CO_2_.

### (b) Methane removal model intercomparison project

Earth System Modelling and experiments can help to quantify the expected impacts of methane removal, particularly through the development of methane emissions-driven models that include interactive chemistry and carbon cycles. These would include the dependence of methane lifetime on methane itself and on other ozone precursors and allow for climate change feedbacks on methane lifetime and natural methane emissions from permafrost soils and wetlands. These feedbacks could affect the climate benefits of methane removal compared to those of CDR.

We recommend a Methane Removal Model Intercomparison Project to provide structure for a multimodel analysis. Similar to and inspired by previous analyses and intercomparisons for CO_2_ (e.g. [28,103]), a full investigation for methane removal is needed to examine:

(1) Scenarios of different timing and amounts of methane removal;
(2) Comparisons of the climate impacts and Earth-system feedbacks of methane removal in different atmospheric and climate scenarios (e.g. low- and high-emission);
(3) Spatially explicit simulations of methane removal at prescribed locations and latitudes (requiring models to have an ‘emissions-driven’ methane capability);
(4) Studies of how methane's relatively short lifetime, in conjunction with climate feedbacks on natural methane emissions, influences metrics of cumulative methane removal;
(5) Feedbacks with air quality, including tropospheric ozone (O_3_) concentrations, through OH chemistry and/or secondary aerosol formation;
(6) Interactions of methane removal with other mitigation and CDR approaches.

Studies are also needed to examine the impacts of methane removal beyond temperature and air quality by quantifying the consequences of different negative emissions technologies in terms of their land, water, and energy requirements and investment costs, as done previously for CO_2_ (e.g. [33,37,104]). Such an analysis would allow a more direct comparison between various greenhouse gas removal technologies, which could then be evaluated using integrated assessment models.

## 4. Discussion

Methane removal is a complement to, not a replacement for, mitigating methane and carbon dioxide emissions. If methane removal proves feasible and deployable at scale, methane's relatively large GWP over the first few decades could provide advantages compared to CO_2_ mitigation in slowing the near-term rate of global warming [105]. Combined with stringent CO_2_ emissions reductions and removals resulting in a temperature ‘overshoot' scenario, methane removal could also prove valuable for reducing peak temperatures, if it can be scaled sufficiently quickly.

Scenarios of methane removal should be evaluated formally, similar to research for CO_2_ removal, including experiments, modelling and technology development. These efforts should include the Methane Removal Model Intercomparison Project proposed above to examine the climate and Earth-system consequences of different methane removal amounts, locations and timings. Along with regional and global temperature outputs, model outputs could also be examined for changes in the number of extreme weather events, implications for air quality and other Earth-system feedbacks. Integrated assessment models could also be modified to include policy or pricing scenarios of methane removal, similar to evaluations of carbon dioxide removal.

All negative-emission technologies, including those for methane removal, need to be examined through the lens of social considerations that could limit research and deployment. Social pressures contributed to legal restrictions on iron fertilization in the oceans and have curbed deployment of geological carbon storage projects in Germany and the Netherlands [106]. Compared to CO_2_, one advantage of complementary methane removal technologies is that capture and storage are unnecessary, avoiding long-term monitoring costs and potential storage reversals. In California, for example, project operators are required to monitor a CO_2_ injection site for a century after injection to document permanence—that the CO_2_ is retained on site, typically in a geologic storage reservoir [107]. For purposes of verification, oxidizing CH_4_ to CO_2_ should be relatively simple to quantify in input and output gas streams. The main measurements needed are the flow rate of gas through the system and the changes in concentration for CH_4_ and CO_2_ (with occasional monitoring of partial oxidation by-products such as carbon monoxide). The methane offset can be verified in real time.

Another consideration for active methane-removal systems is the volume of air needed to be processed to remove teragrams of methane. If air handling is to be undertaken at large scales, it would make economic sense to convert other greenhouse gases simultaneously, particularly the catalytic reduction of N_2_O to N_2_ [31]. Although our current paper emphasizes methane removal, co-removal of other gases would reduce unit costs.

Currently, few financial incentives exist for large-scale methane or carbon dioxide removal by the private sector. Projects for greenhouse gas capture may therefore require significant public funding, policy mandates [106,108,109], or inclusion in a technology-neutral GHG pricing scheme. Although demonstration projects (e.g. small-scale plants and component testing facilities) can face ‘not in my backyard' opposition locally [110], they can sometimes bolster public support for greenhouse gas removal technologies. However, research is needed on the extent to which demonstration-scale plants for greenhouse gas capture may result in perceived ‘moral hazards', inadvertently reducing support for greenhouse gas emission mitigation [111].

Avoiding methane emissions through local mitigation at point sources will typically be less expensive and more efficient than removing methane from ambient air *after* emissions. However, local mitigation efforts may be insufficient for meeting the target of the Paris Agreement in terms of both scale and speed. Staying below the 1.5 or 2°C warming targets strongly depends on the energy sector, which has considerable system inertia [112]. Energy infrastructure facilities typically operate and are paid for over decades, which makes near-term substitutions of recently deployed energy plants expensive and unlikely [113].

Although existing methane mitigation approaches are needed globally, temperature stabilization by mid-century may also require new greenhouse gas removal technologies. As stipulated in Article 10, paragraph 5, of the Paris Agreement, accelerating, encouraging and enabling innovation is critical for an effective, long-term global response to climate change. Developing these greenhouse gas capture technologies will require technology development, including prototype testing in the laboratory and field. Just as importantly, new models for accelerating innovation and closing knowledge gaps on public acceptance and demand-side innovation are needed, including incentives for early adoption and developing niche markets [114]. Negative emission technologies are expected to become a key tool for climate change mitigation in the second half of the century and may help keep mitigation costs fairly low [112,115].

Finally, research on methane removal is warranted for scenarios where potential temperature increases rise above 2°C. Overshoot of 2°C global surface temperature appears increasingly likely given recent rates of greenhouse gas emissions [5,13,32,116,117]. Such overshoot would require greenhouse gas removal to bring atmospheric levels in line with a given temperature threshold. Beyond anthropogenic emissions, we also cannot ignore the possibility of accelerated methane release from natural systems, such as widespread permafrost thaw or release of methane hydrates from coastal systems in the Arctic. Such Earth-system feedbacks could require methane removal to offset releases even if anthropogenic emissions are reduced substantially.

For many reasons, then, we believe a systematic research program for methane removal is needed today that includes experiments, technology development and modelling. The Methane Removal Model Intercomparison Project proposed here can quantify the global and local impacts of methane removal, and allow for comparisons with carbon dioxide removal, potentially informing policy decisions. Testing of various methane removal technologies and validation at scale will clarify which approaches are most effective, acknowledging the priority of emissions reductions for methane and carbon dioxide.

## Data accessibility

This article has no additional data.
